# Case Report: Significant Response to Immune Checkpoint Inhibitor Camrelizumab in a Heavily Pretreated Advanced ER+/HER2− Breast Cancer Patient With High Tumor Mutational Burden

**DOI:** 10.3389/fonc.2020.588080

**Published:** 2021-02-09

**Authors:** Rong Wang, Yuchen Yang, Wei-Wu Ye, Jianxing Xiang, Songan Chen, Wei-Bin Zou, Xiao-Jia Wang, Tianhui Chen, Wen-Ming Cao

**Affiliations:** ^1^ Department of Breast Medical Oncology, Cancer Hospital of the University of Chinese Academy of Sciences (Zhejiang Cancer Hospital), Hangzhou, China; ^2^ Institute of Cancer and Basic Medicine (IBMC), Chinese Academy of Sciences, Hangzhou, China; ^3^ Burning Rock Biotech, Guangzhou, China; ^4^ Department of Cancer Prevention, Cancer Hospital of the University of Chinese Academy of Sciences (Zhejiang Cancer Hospital), Hangzhou, China

**Keywords:** breast cancer, immunotherapy, biomarker, TMB, NGS, molecular monitoring

## Abstract

Endocrine treatment plus CDK4/6 inhibitors have become standard of care for estrogen receptor positive (ER+) breast cancer. Although immune checkpoint inhibitors (ICIs) have shown promising antitumor activity in a variety of cancer types, only limited success has been achieved for metastatic breast cancer (mBC) patients, especially the ER+ subtype, which usually exhibit lower tumor mutation burden (TMB) compared with other subtypes and therefore perceived as immunologically quiescent. Here we present a case of an ER+/HER2- but TMB-high mBC patient who had significant response to combination therapy with anti-PD-1 antibody camrelizumab and vinorelbine and obtained partial response (PR) with a progression-free survival (PFS) of 5 months after failure of multiple lines of therapy. Our case indicates that TMB may serve as a potential biomarker in immunotherapy selection for normally immunologically “cold” tumors such as ER+ mBC, also molecular monitoring during the whole treatment course plays an important role in patient management.

## Introduction

Breast cancer is the most common cancer worldwide for females, accounting for almost a quarter of all female cancers (1). There are three main molecular subtypes in breast cancer: hormone receptor-positive (HR+), human epidermal growth factor receptor-2 amplified/overexpressed (HER2+), and triple-negative (TN). HR+ tumors include ER+ and/or progesterone receptor-positive (PR+) breast cancers. ER+ subtypes are the main group of breast cancers, accounting for 75% (2). Within this population, traditional endocrine therapies targeting estrogen receptor such as tamoxifen, fulvestrant, and aromatase inhibitors (AI) have being widely used clinically. About 30% patients acquired resistance during endocrine treatment (3) and drugs targeting on cell-signaling pathways such as CDK4/6 inhibitor has been recognized to relieve resistance. CDK4/6 inhibitor palbociclib in combination with letrozole prolonged PFS for almost 10 months in ER+ HER2- mBC compared with placebo plus letrozole in study PALOMA-2 (4). Also, study PALOMA-3 showed that fulvestrant plus palbociclib was associated with significant improvement in PFS compared with fulvestrant plus placebo (5).

Besides endocrine and targeted therapies, ICIs such as PD-1 and programmed cell death 1 ligand 1 (PD-L1) inhibitors have revolutionized cancer treatment in recent years. ICIs exert anti-tumor activity mainly through enhancing host immune response. However, only a small fraction of metastatic breast cancer (mBC) patients have benefited from ICIs. Atezolizumab plus nab-paclitaxel has been approved for triple-negative breast cancer (TNBC) with ≥1% PD-L1 expression on tumor-infiltrating immune cells based on the IMPASSION130 study (6). Also, TMB was assumed to create neo-antigens, which could exert host immune response. Thus, TMB has become an increasingly important biomarker in immune treatment. However, one of the challenges for immunotherapy in mBC is that breast tumors have always been perceived as immunologically quiescent with lower TMB levels compared with NSCLC and melanoma (7). TMB varies in the subtypes of breast cancers, with HER2+ and TNBC exhibiting higher burden than HR+ subtypes (8, 9).

In the current report, we present a case of an ER+/HER2-, TMB-high mBC patient who had significant response to an anti-PD-1 antibody Camrelizumab after failure of multiple lines of therapy. This case report will provide new insights into TMB level and corresponding immunotherapy strategy of ER+ mBC in clinical practice. TMB level might serve as the potential biomarker for the efficacy of ICIs, and molecular monitoring during the whole course plays an important role in patient management.

## Case Presentation

**Figure 1:**
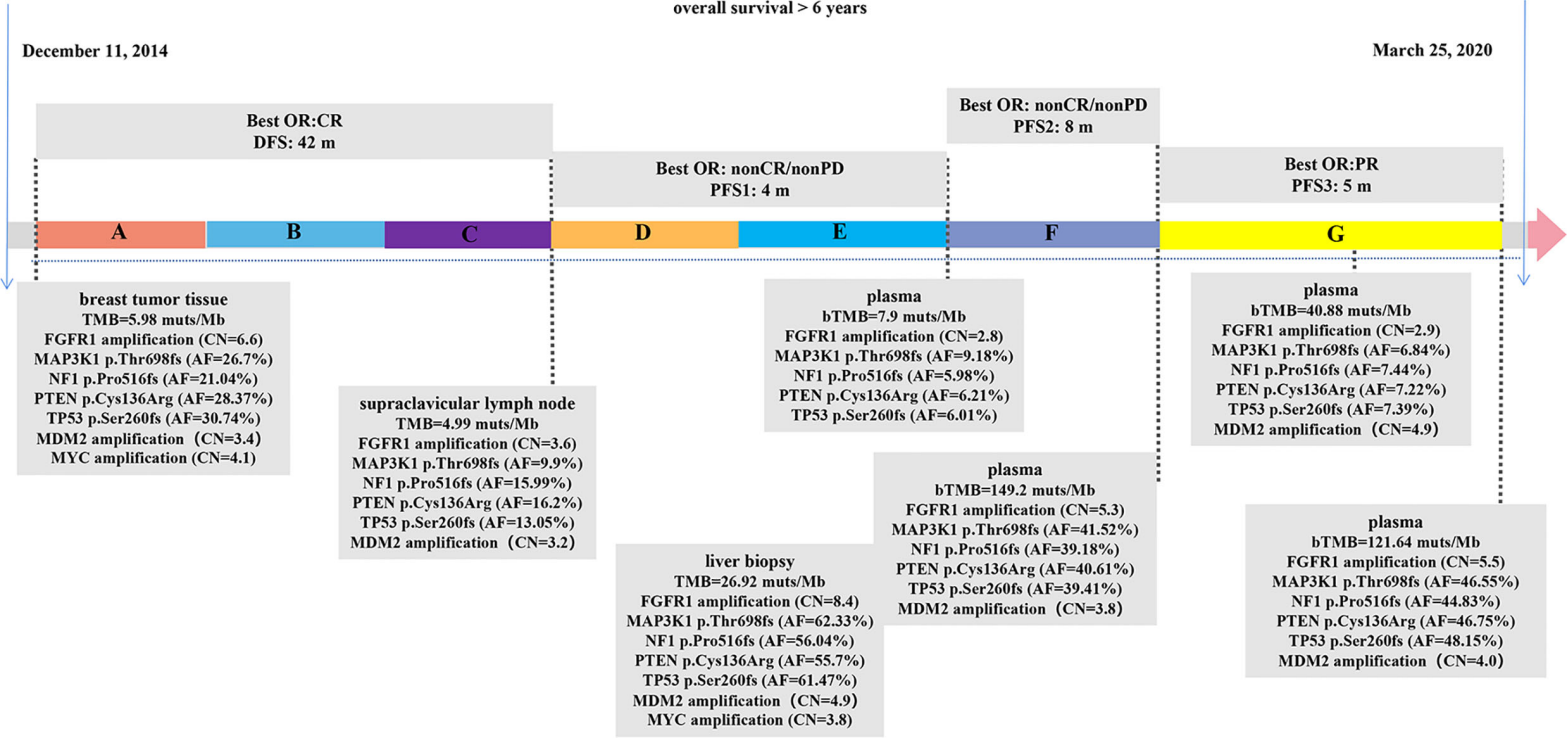
Therapeutic course timeline followed in the patient. **(A)** Doxorubicin+Cyclophosphamide+Docetaxel (AC-T), **(B)** Tamoxifen, **(C)** Anastrozole, **(D)** Fulvestrant, **(E)** Fulvestrant+Palbociclib, **(F)** Albumin paclitaxel+radiotherapy, **(G)** Camrelizumab+Vinorelbine. bTMB, blood TMB; CR, complete response; PR, partial response; PD, progressive disease; DFS, disease-free survival; PFS, progression-free survival; AF, allelic frequency; CN, copy number.

The patient management is described in **Figure 1**. A 57-year-old female patient who underwent a right breast modified radical mastectomy in December 2014 with the post-operative pathologic diagnosis as grade 3 invasive breast cancer without lymph node metastasis, pT1N0M0, stage IA. The results of immunohistochemistry (IHC) were positive ER (ER+), negative progesterone receptor (PR-), no HER2 overexpression (IHC 0), and a Ki67 level of 15%. After surgery, the patient received adjuvant chemotherapy of doxorubicin 60 mg/m^2^ and cyclophosphamide 600 mg/m^2^ once every 21 days for four cycles followed by docetaxel 80 mg/m^2^ once every 21 days for four cycles (AC-T regimen). After chemotherapy, she began adjuvant endocrine therapy of tamoxifen for 2 years and anastrozole for one year. In the end of July 2018, she was admitted to hospital due to the lump detected in the neck for 20 days, and the initial supraclavicular lymph node biopsy suggested metastasis. The corresponding IHC results displayed as positive ER at 20–30%, PR- and HER2 (1+). Subsequent positron emission tomography-computed tomography (PET-CT) showed bone metastasis and multiple lymph nodes in the whole body.

The patient began fulvestrant endocrine therapy, at the dose of 500 mg on day 0, 14, 28, and every 28 days thereafter, with zoledronic acid targeting on bone-related events soon after the progression detected. CDK4/6 inhibitor palbociclib (125 mg orally every day) was also added after one-month treatment as it was listed on September 2018 in China. However, computed tomography (CT) showed progressive disease (PD) in multiple lesions of coelom lymph nodes, producing a PFS of 4 months. Circulating free DNA (cfDNA) from patient’s plasma was subjected to next generation sequencing (NGS) using OncoScreen Plus panel (Burning Rock Biotech, Guangzhou, China). NGS revealed an amplification of *FGFR1*, mutations in *MAP3K1, NF1, PTEN, TP53*, which were all presented in tumor tissue samples from surgery and supraclavicular lymph node biopsy assessed by the same NGS panel (**Figure 1**, **Supplementary Table 1**). The blood TMB (bTMB) from the plasma was 7.9 mutations/megabase [mut/Mb], tissue TMB (tTMB) detected from surgery and supraclavicular lymph node biopsy were at modest levels of 5.98 mut/Mb and 4.99 mut/Mb respectively.

Albumin paclitaxel monotherapy was then administered at the dose of 125 mg/m^2^ for day 1, 8, and 15 every 4 weeks, yielding a best response of non-complete response (CR)/non-PD, with a PFS of 8 months. In detail, approximately six months after chemotherapy administration, the patient received radiotherapy with 30Gy/10F to the brain because magnetic resonance imaging (MRI) displayed multiple metastatic parenchyma in both sides. After another month, both CT and upper abdominal MRI showed metastasis in multiple lesions in liver, which were pathologically defined as poorly differentiated carcinoma through coarse needle biopsy with positive ER at 20%, PR-, no HER2 overexpression (IHC 0), and a Ki67 level of 30%. Liver biopsy sample and cfDNA from plasma were both evaluated using OncoScreen Plus NGS assay. NGS showed extremely high TMB in both samples: 26.92 mut/Mb in tumor lesion and 149.2 mut/Mb in cfDNA.

**Figure 2:**
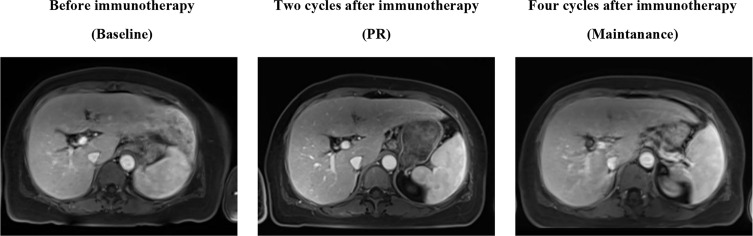
MR Image scans revealed the clinical response to the treatment of PD-1 antibody camrelizumab combined with vinorelbine.

**Figure 3:**
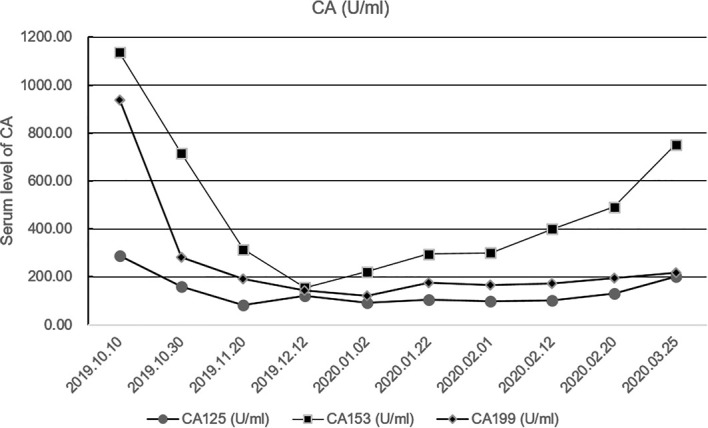
Fluctuation of CA level during immunotherapy in the blood including CA125, CA153, CA199. CA, carbohydrate antigen.

**Figure 4:**
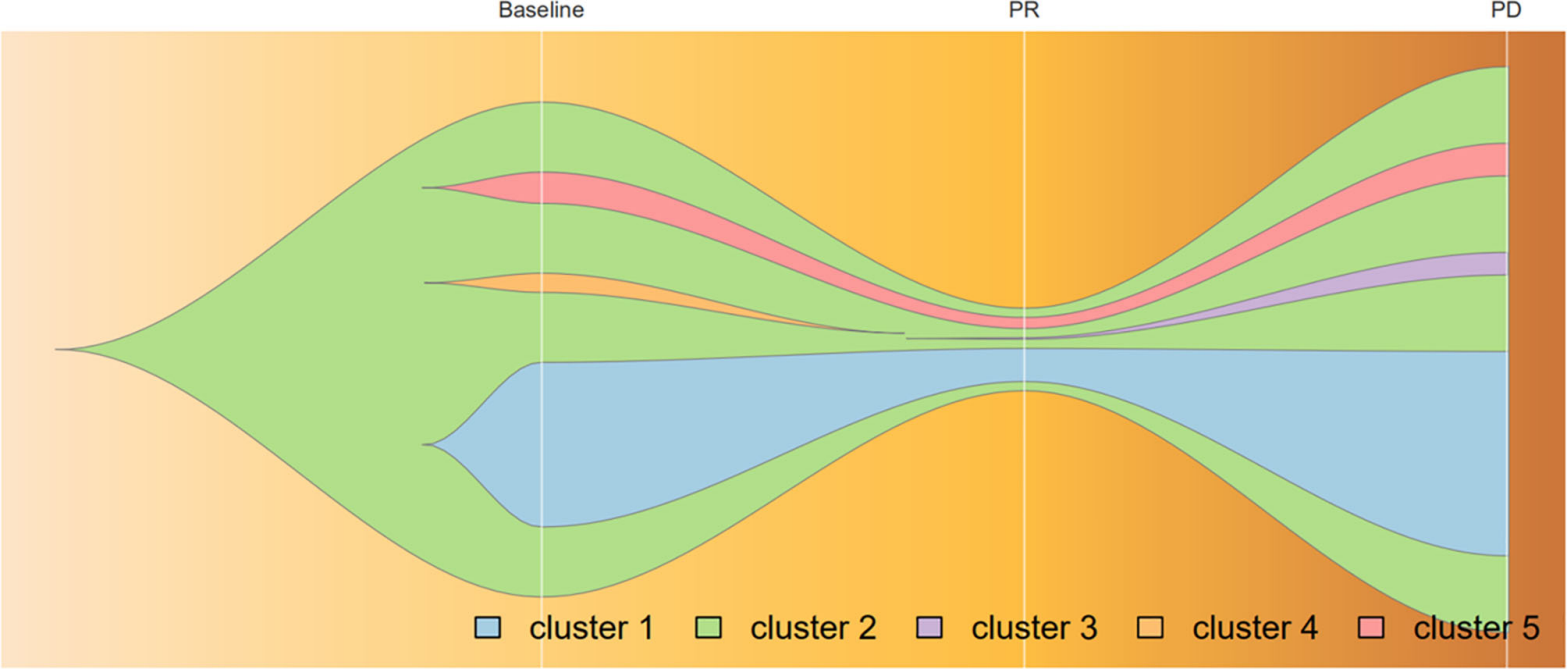
Progression of cfDNA mutation burden during camrelizumab treatment, analyzed by variant allele frequencies. Frequencies of variants in Cluster 1 and 2 fluctuated along with treatment effectiveness, while those in Cluster 5 remained steadily; Mutations in cluster 3 acquired as disease progression and those in cluster 4 vanished before PR achieved. Broken vertical white lines correspond to plasma sample before immunotherapy, PR, and relapse subsequent to treatment.

Because of the high TMB detected, camrelizumab (at the dose of 200 mg for day 1 every 3 weeks) combined with vinorelbine (40 mg for day 1 and day 8) were innovatively attempted after NGS testing on September 28, 2019. After two cycles of treatment, MRI displayed that the intrahepatic lesions had markedly shrunken in size and maintained after four treatment cycles (**Figure 2**), also carbohydrate antigen (CA) level in the blood decreased rapidly during the first 4-cycle treatment (**Figure 3**), yielding the best response of partial response (PR). NGS of plasma cfDNA performed after two cycles of treatment showed a decrease in bTMB level from 149.2 to 40.88 mut/Mb, also other plasma cfDNA mutations were divided into 5 clusters according to their frequency referred to the computational method SciClone (10) and frequencies fluctuated accordingly during immunotherapy (**Figure 4**), suggesting that treatment had been effective from the molecular aspect. However, the patient experienced PD on March 25, 2020, with a PFS of 5 months. During the course of camrelizumab treatment, the patient developed grade 2 adverse effects of cutaneous capillary endothelial proliferation (CCEP), mainly displayed in neck and fingers, but the patient was able to tolerate the treatment without any dose reduction.

## Discussion

Recent successful development of immunotherapy with ICIs for cancer treatment has rekindled enthusiasm for the research of immune system. Various agents of ICIs such as inhibitors of cytotoxic T-lymphocyte–associated antigen (CTLA-4), PD-1, and PD-L1 have been approved by FDA in several solid tumors and classical Hodgkin lymphoma. For example, PD-1 antibody pembrolizumab has been approved in many tumor types including NSCLC, SCLC, melanoma, and refractory solid cancers with microsatellite instability. As is mentioned above, atezolizumab plus nab-paclitaxel has been approved for TNBC, as the only approved agent in breast cancer. Recently, a few clinical trials targeting on HR+ breast cancers were launched but the results so far are not promising. In a phase Ib trial JAVELIN (11), PD-L1 antibody avelumab was evaluated for all subtypes of mBC, ORR was only 2.8% for unselected HR+/HER2- cohort. In another phase Ib trial KEYNOTE-028 (12), efficacy of pembrolizumab for PD-L1 positive ER+/HER2- mBC was assessed, ORR was 12% with 1.8-month PFS and 8.6-month OS.

Somatic mutations presented in cancer genome lead to the creation of neo-antigens, which serve as the target for anti-tumor immune activity (13, 14). In another word, the high level of mutation burden a tumor possesses could be associated with the production of high level of neo-antigens, which will present and be recognized by the immune system. Consistently, many types of tumors such as NSCLC and melanoma have shown high TMB level and it presents as the predictor of improved response, durable clinical benefit and PFS to ICIs in such tumors nowadays (15, 16). However, breast cancer has always been treated immunologically as “cold” tumors, because such tumors are less immunogenic and the general TMB level could be much lower, especially for HR+ subgroups (7–9). Keynote 119 study displayed that mTNBC patients with high TMB were associated with better clinical benefit with pembrolizumab compared with chemotherapy (17). In addition, analysis from other studies demonstrated that breast cancers including TNBC and other molecular subtypes with high TMB could also benefit from immunotherapy (9, 18). Patient in our case displayed high TMB in liver metastatic lesions and cfDNA in plasma and received ICIs treatment camrelizumab in combination with chemotherapy accordingly. Camrelizumab is an anti- PD-1 monoclonal antibody, which has been approved for the indication of relapsed/refractory classical hodgkin lymphoma and advanced hepatocellular carcinoma by National Medical Products Administration (NMPA) in China. Also, bTMB levels of the patient showed obvious decrease after two months’ ICIs treatment (from 149.2 to 40.88 mut/Mb) and remained stable for several months, which may note us that bTMB could be a potential monitoring biomarker of ICIs treatment.

Approximately 75% breast cancers have been defined as ER+ breast cancers (2). Interfering with hormone estrogen action such as tamoxifen has become a main therapy in this cohort for a long time (19). While drug resistance to endocrine treatment is not rare in ER+ mBC patients and various endocrine resistance mechanisms have been recognized, such as loss of ERα expression, increasing expression of signaling pathway such as CCND1-CDK4/6-RB and other biological processes (20). CDK4/6 inhibitor palbociclib displayed great antitumor efficacy with hormonal therapies. As is mentioned, in the phase III PALOMA-2 study, palbociclib combined with letrozole resulted in longer PFS than that with letrozole alone for previously untreated ER+ breast cancers (PFS: 24.8 vs. 14.5 mon, hazard ratio 0.58; 95% CI, 0.46 to 0.72; P < 0.001) (4). Subsequent phase III PALOMA-3 study further demonstrated that for ER+ breast cancers who had progressed on previous endocrine therapy, palbociclib along with fulvestrant resulted in longer PFS than fulvestrant alone (PFS: 9.5 vs. 4.6 mon, hazard ratio 0.46, 95% CI 0.36–0.59, p < 0·0001) (21, 22).

Genomic aberrations in patients from PALOMA-3 (23) were further analyzed, *FGFR1* amplification was found in patients at risk of early progression. A recent clinical trial found out that patients with *FGFR1* amplification were more likely to be the PR- subtype and experienced shorter time to progression with both endocrine therapy and in combination with CDK4/6 inhibitor (24). In addition, *FGFR1* amplification has been recognized as a resistance mechanism for the treatment of CDK4/6 inhibitor plus fulvestrant in ER+ breast cancer cells, also NGS results of ctDNA from patients enrolled in MONALEESA-2 showed that patients with *FGFR1* amplification exhibited a shorter PFS compared to patients with wild-type *FGFR1* under the treatment of CDK4/6 inhibitor plus letrozole (PFS: 10.61 vs. 24.84 mon, p = 0.075) (25). In our case, *FGFR1* amplification was detected in tumor specimens from surgery and remained in ctDNA along with the treatment, which may help explain the short PFS of only 4 months with CDK4/6 inhibitor palbociclib plus fulvestrant.

To our knowledge, this is the first case to demonstrate the anti-tumor activity of PD-1 antibody camrelizumab in heavily treated ER+ mBC with high TMB. In this case, molecular monitoring through NGS platform along with treatment provided useful information regarding drug efficacy and selection. NGS panel assessed TMB may help define a patient group which could potentially benefit from immune treatment, even in ER+ mBC commonly known as immunosuppressive. Moreover, assessment of bTMB levels may also aid in evaluating ICIs efficiency in the course of drug usage.

## Conclusions

In conclusion, we present a case of combination therapy of PD-1 antibody camrelizumab and chemotherapy for an ER+ breast cancer patient with high TMB who developed resistance to multiple lines of therapy. Camrelizumab combination therapy achieved PR with a PFS of 5 months. We therefore propose that TMB may serve as a potential biomarker in immunotherapy selection for normally “cold” tumors such as ER+ mBC.

## Ethics Statement

The studies involving human participants were reviewed and approved by the ethnic committee of the Cancer Hospital of the University of Chinese Academy of Sciences (Zhejiang Cancer Hospital). The patients/participants provided their written informed consent to participate in this study. Written informed consent was obtained from the individual(s) for the publication of any potentially identifiable images or data included in this article. 

## Author Contributions 

RW, WC, and SC identified the case. RW, WY, WZ, XW, and TC collected the clinical information, diagnostic information, therapeutic information, and images of the patients. YY wrote and submitted the manuscript. JX revised the manuscript. WC and RW proofread the manuscript. All authors contributed to the article and approved the submitted version.

## Funding

This study was supported by grants from the Natural Science Foundation of Zhejiang Province, China (grant number: LY17H160038), Key Research-Development Program of Zhejiang Province (grant number: 2020C04012, 2019C04001, 2017C03013), and Science and Technology Program offered by the Health Bureau of Zhejiang Province, China (grant number: 2017RC014).

## Conflict of Interest

YY, JX, and SC were employed by the company Burning Rock Biotech.

The remaining authors declare that the research was conducted in the absence of any commercial or financial relationships that could be construed as a potential conflict of interest.

## Abbreviations

AI, aromatase inhibitors; CA, carbohydrate antigen; CCEP, cutaneous capillary endothelial proliferation; cfDNA, circulating free DNA; CR, complete response; CT, computed tomography; CTLA-4, cytotoxic T-lymphocyte–associated antigen; ER+, estrogen receptor positive; HER2+, human epidermal growth factor receptor-2 amplified/overexpressed; HR+, hormone receptor-positive; ICIs, immune checkpoint inhibitors; IHC, PET-CT: immunohistochemistry; mBC, metastatic breast cancer; MRI, magnetic resonance imaging; NGS, next generation sequencing; NMPA, National Medical Products Administration; PD, progressive disease; PD-1, programmed cell death receptor 1; PD-L1, programmed cell death 1 ligand 1; PET-CT, positron emission tomography-computed tomography; PFS, progression-free survival; PR, partial response; PR+, progesterone receptor-positive; PR-, negative progesterone receptor; TMB, tumor mutation burden; bTMB, blood TMB; tTMB, tissue TMB; TN, triple-negative; TNBC, triple-negative breast cancer.
